# The Effect of Using Assessment Instruments on Substance-abuse Outpatients' Adherence to Treatment: a Multi-centre Randomised Controlled Trial

**DOI:** 10.1186/1472-6963-11-123

**Published:** 2011-05-25

**Authors:** Veerle Raes, Cor AJ De Jong, Dirk De Bacquer, Eric Broekaert, Jan De Maeseneer

**Affiliations:** 1 De Sleutel, Dept. of Research and Quality Assurance, Jozef Guislainstraat 43a, 9000 Ghent, Belgium; 2 Nijmegen Institute for Scientist-Practitioners in Addiction, Radboud University Nijmegen, Postbus 9104, HE Nijmegen; Novadic-Kentron, Network for Addiction Treatment Services, The Netherlands; 3 Department of Public Health, Ghent University, De Pintelaan 185, 9000 Gent, Belgium; 4 Department of Special Education, Ghent University, H. Dunantlaan 2, 9000 Gent, Belgium; 5 Department of Family Medicine and Primary Health Care, Ghent University, De Pintelaan 185, 9000 Gent, Belgium

## Abstract

**Background:**

Drop-out is an important problem in the treatment of substance use disorder. The focus of this study was to investigate the effectiveness of within treatment assessment with feedback directly to patients with multiple substance use disorder on outpatient individual treatment adherence. Feedback consisted of personal resources' and readiness to change status and progress that facilitate or hinder change, thereby using graphical representation.

**Methods:**

Informed consent was obtained from both the control and experimental groups to be involved in research and follow-up. Following Zelen's single consent design, baseline participants (n = 280) were randomised (sample-size-estimation: 80%power, p=.05, 2-sided) and treatment consent was obtained from those allocated to the experiment (n = 142). In both groups, equal numbers of patients did not attend sessions after allocation. So, 227 persons were analyzed according to intention-to-treat analysis (ITT: experiment n = 116;control n = 111). Excluding refusals 211 participants remained for per-protocol analysis (PP: experiment n = 100; control n = 111), The study was conducted in five outpatient treatment-centres of a large network (De Sleutel) in Belgium. Participants were people with multiple substance use disorder -abuse and dependence- who had asked for treatment and who had been advised to start individual treatment after a standardised admission assessment with the European Addiction Severity Index.

The experimental condition consisted of informing the patient about the intervention and of subsequent assessments plus feedback following a protocol within the first seven sessions. Assessments were made with the Readiness to Change Questionnaire and the Personal Resources Diagnostic System. The control group received the usual treatment without within treatment assessment with feedback. The most important outcome measure in this analysis of the study was the level of adherence to treatment at and beyond eight sessions.

**Results:**

Individual treatment that included assessment with feedback increased adherence to treatment at and beyond eight sessions (RR = 1.6,95%CI:1.2-2.2). Benefit was also found at and beyond twelve sessions, which was the number of sessions required to complete 90% of the assessments with feedback in practice (RR = 1.6,95%CI:1.2-2.5).

**Conclusions:**

Assessment with feedback in routine practice improved adherence to treatment. More research is needed to evaluate progress in social functioning and motivation to change in outpatient treatment of substance use disorder, thereby using objective measures

**Trial registration:**

Current Controlled Trials ISRCTN65456186

## Background

Drop-out is an important problem in the treatment of people with multiple substance use disorder. There is growing agreement for substance abuse and dependence, being a chronic and relapsing condition [1-3]. The effectiveness of treatment to stabilize or overcome a chronic condition is strongly related to treatment compliance [3]. Among alcohol-dependent people, diabetics or persons with hypertension it is common to find a compliance rate below 50% [4].

In a therapeutic community environment for drug addicted people, evidence has been found that family-[5,6], social network- [7,8], and staff-involvement [9,10], improve retention levels. In the broader area of health care, patient-based measures in routine practice combined with feedback were found to improve significantly the process and outcome of patient care [11]. In mental healthcare, feedback to clinicians about patients' progress based on routine outcome monitoring affected outcome and even the number of sessions in psychological interventions [12,13].

In the realm of outpatient treatment of substance use disorder, compliance is also associated with session attendance a/o adherence to treatment. Regular treatment often relies on assessment, but outside its use in planning, monitoring, and evaluating interventions, assessment and feedback as part of the treatment itself and its positive effect on patient adherence is under-investigated. Kypri et al. focused on this issue, related to contamination of clinical trials [14], and found that routine screening and minimal assessment themselves may produce some benefit [15]. In line with the studies based on routine outcome monitoring in psychotherapy [12,16], Hawkins et al. [17] developed a similar approach in the treatment of substance use disorder. Their focus was on feedback to clinicians about lack of progress in order to reduce the risk of patient drop-out.

In this study, assessment and feedback is introduced as part of the treatment process itself. It is aimed to offer opportunities to support counselling by establishing a collaborative relationship and a patient-centred focus [18,19]. This therapeutic method of assessment [20] seeks to integrate key features of therapeutic alliance between clinician and patient in assessment and feedback sessions. Feedback is given to the patient each time at the next session after assessment. It consists of communicating the status of substance use, readiness to change, and personal resources that may facilitate or hinder change. Repeated assessment and graphical representation is considered to provide opportunities for feeding back of progress, treatment recommendation, and further action. It is also providing the patient with new ways of thinking and feelings about self and others.

This study addresses the question whether continuing rounds of assessment with personal feedback to patients that replace a number of regular outpatient sessions in the treatment of people with multiple substance use disorder, improves adherence, compared to an outpatient approach without such within treatment assessment and feedback. It is the most important aim of this study to investigate the effectiveness of within treatment assessment and personal feedback to patients on outpatients' adherence to treatment. The reporting follows the CONSORT-guidelines [21-23].

## Method

### Study Design

In an attempt to determine whether enhanced adherence could be demonstrated by the introduction of within treatment assessment and personal feedback in outpatient treatment of substance use disorder, we set up an experimental study. This study was a multi-centre randomised controlled trial registered in the ISRCTN-database (ISRCTN65456186). It was embedded within an already existing system to ask informed consent to all patients for the use of admission assessment and treatment data for research purposes, and for follow-up. To minimize problems associated with the clinicians' resistance to enter patients into a clinical trial, Zelen's single consent design was chosen [24-26]. The key characteristic of this method is that participants were randomised and treatment consent was obtained from participants who were allocated to the experimental intervention, while controls did not receive the experimental treatment but get the best usual care. In such an open trial, statistical power can be affected by a high proportion of participants getting usual care. Therefore, Adamson et al. [27] advised performing sample-size-estimation before the start of the project and an intention-to-treat analysis.

### Setting and Participants

All persons who entered one of the five outpatient drug-treatment centres of the treatment network 'De Sleutel' in Belgium between March 2007 and March 2009 were candidates for inclusion in the study. Based upon yearly reports, patient characteristics in the five centres were assumed to be very comparable. To be taken in charge for treatment in one of these centres, patients should be diagnosed DSM IV substance abuse or dependence for at least one substance, exclusive single alcohol abuse or dependence. The inclusion criteria for the trial were that the patient: (1) gave informed consent about the use of data and being contacted for follow-up, (2) sufficiently understood the Dutch language, (3) passed the full admission assessment, consisting of at least a first contact session, the EuropASI-interview and a feedback session, and (4) was advised to start individual treatment. The study was approved by the Central Ethics Board for the Mental Health Sector of the n.p.o. Provincialat of the Brothers of Charity (ref. OG054-2006-19).

### Assessment instruments

#### EuropASI and feedback form

Admission assessment was based upon the European version of the Addiction Severity Index (EuropASI) [28]. The Dutch version of EuropASI (Cronbach's alphas > =.70, except for employment status) [29], adapted for Flanders, was used. This semi-structured interview offers an inventory of problems in seven potential problem areas (physical health, education-work-income, alcohol use, drug use, judicial, relations, psycho-emotional). The information from ASI is synthesized on a two-sided feedback form, placing positive aspects of the patient's experience against the problems in each life area. The feedback form helps clinicians to communicate findings from EuropASI with the patient and to suggest and support treatment options [30].

#### Readiness to Change Questionnaire (RCQ)

The RCQ was used for within treatment assessment and feedback. Originally, the RCQ was a self-rating questionnaire [31] used to assess the first three stages of change, which are pre-contemplation, contemplation and action. The Dutch version of this instrument [32] has been adapted for poly-drug abusers. For each substance separately, being in action could be distinguished from not being in action (Cronbach's alphas > = .70, except for pre-contemplation) [33]. Feedback was based on the principles from the trans-theoretic model approach. For each substance that was used in the last 30 days, the clinician commented on whether the person was in pre-, contemplation or in action. The information about stage of change was further elaborated, making use of the number and type of used substances, and the number of use-days.

#### Personal Resources Diagnostic system (PREDI)

Two scales were used within treatment from PREDI [34], originally a German instrument consisting of three scales: personal resources and wish to change. The personal resources scale provided the appreciation of both the clinician and the patient, the wish to change scale only the patient's appreciation in 16 important life-areas, which were: every day life situation, living situation, financial situation, legal situation, work situation, health status, health behaviour, substance use, self-esteem, self-realization, self-control, contact with reality, partner relation, family relations, social relations, social cultural situation. It was translated into Dutch, using common rules for cross-cultural adaptations of health measurements [35]. Preliminary to this study, the system was validated in a small sample of patients with substance use disorder (personal resources' Cronbach's alpha=.81; wish to change's Cronbach's alpha=.83) [36]. Feedback consisted of the synthesis of a patient's particular personal resources and a focus on the life areas where the person clearly indicated a wish to change.

### Study interventions

The trial was presented in the centres during the first quarter of 2007. Clinicians were mainly social, educational, and/or psychological workers or psycho-therapists. Their basic training was completed with EuropASI-assessment and feedback, and with motivational interviewing. They had at least one year of training or experience. Inspired by Del Boca & Darkes' guidelines [37] for enhancing validity and utility of randomised controlled trials, a manual was devised that included a training DVD for the experimental sessions. Extra training for supplementary assessment (RCQ and PREDI) with feedback took place in the centres between April and June 2007.

The admission assessment was similar in all centres for all patients. This study focused on the individual treatment after admission, where the treatment length usually was open-ended. Regular treatment consisted of non-manualized individual counselling sessions of about one hour, principally aimed at changes in drug-use behaviour. The expected treatment duration, number or intensity of counselling sessions were defined only by the clinicians' subjective judgment about progress towards goals and life changes, although such progress was not measured objectively.

In the experiment, continued assessment and feedback sessions according to the manual, replaced the regular treatment sessions (Table 1). The therapists themselves administered the assessments and/or gave feedback within the one-hour-sessions instead.

**Table 1:** Scheduling of planned assessment and feedback sessions

session number	experimental group planned assessment and feedback	control group
1	information plus consent First Readiness to Change Questionnaire (RCQ)	Regular session
2	First RCQ (1)	Regular session
3	Feedback on first RCQ	Regular session
4	Regular session	Regular session
5	Personal Resources Diagnostic system (PREDI), part one	Regular session
6	PREDI, part two	Regular session
7	Feedback on PREDI	Regular session

8	Regular session	Regular session
9	Second RCQ (± 30 days after first RCQ)	Regular session
10	Feedback on second RCQ	Regular session
11	Regular session	Regular session

12	...	...

(1) in some cases the First RCQ was completed at the same session where informed consent was given, in other cases at the next session

Unlike those in the control group, people in the experimental group were informed in the first session about further sessions dealing with continued assessment and feedback and asked for consent. During session three (Table 1), the clinician provided the patient with feedback on RCQ using the trans-theoretic model, graphical representation of change and worksheets [38]. For feedback on PREDI a standardised form was used that showed a time-schedule and the personal resources on which to focus further actions. Planning for repeated measurement with feedback using the same instruments was left to the clinicians, with about 30 days in between as a guideline. The researcher closely followed up the clinicians' adherence to the manual.

### Outcome measure

Data-collection on patients' attendance at counselling sessions was part of the routine-administration in the central database of the De Sleutel-network. The start and end date as well as the clinicians' evaluation at the end of the whole treatment was also documented, but the most important outcome measure in this study was the adherence to individual counselling treatment. Adherence was measured by counting the total number of sessions between the date of the first session after admission and the last session date. Therefore, the initial assessment sessions being part of the admission assessment did not count towards sessions in the outcome measure. Since the first five trial related assessment and feedback sessions in the experimental group take approximately seven sessions (Table 1), completing at least eight individual counselling sessions after admission was set as a first outcome measure. In reality, however, 90% of the assessment and feedback activities took place within the first twelve sessions, so completion of at least twelve sessions was set as a second outcome measure.

### Sample size and power

**Figure 1:**
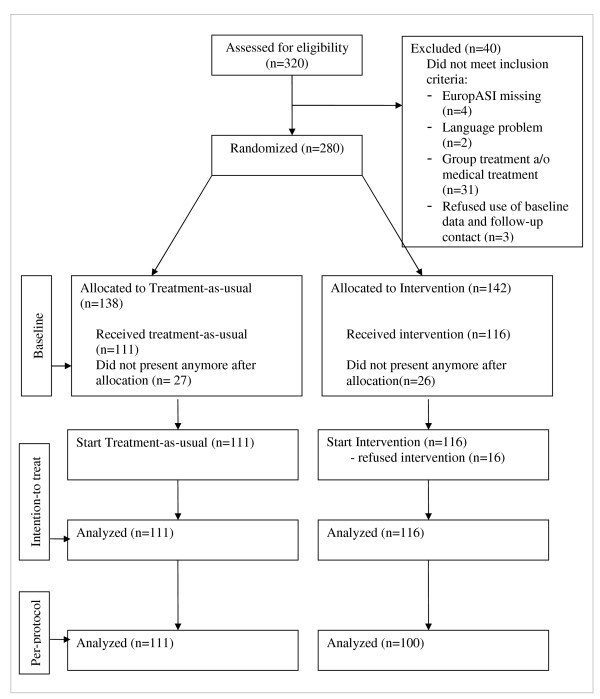
**Flow-chart of patient-acceptance process for the trial**.

Sample size estimation was based upon the results of a study on retention in treatment, retrieved from the central database of the De Sleutel-network during the period from 1999 to 2006. The usual course of treatment took six months for 80% of the patients during that period, with a mean of 5.9 sessions (± 3.5). 56% of patients had fewer than five sessions, 18.3% had eight or more sessions. Based upon 80% power to detect statistically significant differences (p=.05;two-sided), a sample size of at least 100 patients in each study arm was required to demonstrate doubling of the number of patients attending eight or more sessions. While power calculations yield the number of subjects needed at the end of follow-up, we aimed at selecting more subjects (n = 320) and accounted for likely drop-out [39] (Figure 1).

### Randomisation and enrolment

The researcher distributed five closed envelopes to each outpatient centre each month - two experimental/three controls or three experimental/two controls. Each of the closed envelopes was assigned to an eligible patient by the clinical supervisor, and only after the assignment was made the envelope was opened to determine whether the patient was assigned to the control group or the experimental group. The researcher evaluated monthly the enrolment process and confirmed whether the patients assigned met or did not meet all eligibility criteria.

320 patients were assessed for eligibility, of which 40 were excluded because they did not meet the inclusion criteria, so at baseline 280 persons were randomly assigned to the experimental group (n = 142) and to the control group (n = 138) (Figure 1). 26 eligible patients assigned to the experimental group and 27 patients assigned to the control group and being referred to individual treatment did not attend any sessions after admission assessment. They were excluded from further analyses. Thus, the intention-to-treat analysis is based on 116 patients in the experimental group and 111 patients in the control group. Of them who attended their first individual counselling session, 16 patients in the experimental group refused participation and were excluded for per-protocol analysis.

### Statistical Analysis

The Pearson Chi-square test and the independent samples T-test were used to test for differences in patient characteristics between both allocations at baseline. A p-value of .05 or less was considered to indicate statistical significance. The outcome measure was adherence to treatment at and beyond eight and twelve sessions. Risk-ratios (RR) with accompanying 95% confidence intervals were calculated to evaluate adherence to the individual treatment that included psychological assessment and feedback. In this study, the word "risk" referred to a desired effect. Risk ratios of 1 indicate a null-finding, while 95% confidence intervals (CI) should not include 1 to be statistically significant. Risk ratios > 1 are indicating enhanced likelihood of the desired effect. All analyses were performed using SPSS for Windows (release 16.0).

## Results

### Baseline characteristics by allocation

To exclude for bias, the characteristics at baseline of patients allocated to the experimental group are compared with those of the control group (Figure 1, first split). Table 2 shows several patient characteristics in both groups. All data were retrieved from EuropASI. No statistically significant differences were found between the groups (p < .05), except for problem-severity concerning work and substance-abuse. In the control group, more patients had severity indexes for work above four and a bit smaller average substance-abuse severity index than the experimental group. The profiles showed that participants were mainly never-married male Belgians with an average age of 27. Most patients lived with parents or alone. Their schooling degree was low with less than 10% who finished secondary school. About two-thirds were employed full-time or part-time. Cannabis was the primary substance of abuse, followed by cocaine, opiates, and amphetamines. For almost half of the study group, the treatment was their first treatment for drugs/substance abuse. Higher problem severity was most frequent in the area of drug abuse, followed by family and social relationships and psycho-emotional issues. No statistically significant difference between the two groups was found in the number of persons that did not present anymore after allocation (Figure 1). These individuals - respectively 27 and 26 persons in the control and the experimental group - were not included in further analyses.

**Table 2:** Sample characteristics at baseline by allocation

	Experimental (n = 142)		Control (n = 138)		Pearson Chi^2 ^or T-value	P	
Mean age ± SD,y	27.4 ± 7.1		26.9 ± 6.6		.59	.55	n.s.

Men, n (%)	120	(84.5)	108	(78.3)	1.8	.22	n.s.

Country of birth, n (%)							
Belgium	131	(92.3)	134	(97.1)	8.3	.49	n.s.
Other	11	(7.7)	4	(2.9)			

Relationship, n (%)							
Never been Married	119	(83.8)	111	(80.4)	3.6	.61	n.s.
Living situation, n(%)							
Partner & children	19	(13.4)	18	(13.0)	9.6	.30	n.s.
Partner no children	23	(16.2)	31	(22.5)			
With parents	45	(31.7)	37	(26.8)			
Alone	35	(24.6)	29	(21.0)			

Educational level, n (%)							
< = vocational until 2nd degree	58	(42.6)	56	(42.1)	7.2	.62	n.s.
Vocational & 3rd or 4th degree	37	(26.1)	30	(21.7)			
Technical secondary	24	(16.9)	20	(14.5)			
General Secondary	8	(5.6)	12	(8.7)			

Employment status, n (%)							
Employed full-time or part-time	94	(68.1)	90	(65.2)	8.8	.26	n.s.
Profession, n(%)							
Unschooled labour	71	(50.0)	52	(37.7)	7.5	.28	n.s.
Schooled labour	41	(28.9)	45	(32.6)			
Other or missing	30	(21.1)	41	(29.7)			

Primary substance, n (%)							
Amphetamines	17	(12.0)	12	(8.7)	17.4	.19	n.s.
Cannabis	38	(26.8)	51	(37.0)			
Cocaine	30	(21.1)	19	(13.7)			
Opiates	30	(21.1)	19	(13.8)			

Mean years of drug use ± SD							
Amphetamines	4.6 ± 5.0		3.3 ± 3.1		1.8	.07	n.s.
Cannabis	8.0 ± 5.8		7.9 ± 5.4		.91	.84	n.s.
Cocaine	3.1 ± 3.7		3.1 ± 3.7		-.10	.92	n.s.
Opiates	3.8 ± 4.1		3.0 ± 2.3		1.0	.30	n.s.

Mean n of drugs ever used ± SD	3.9 ± 2.0		3.7 ± 1.9		.98	.38	n.s.

Mean n of treatments drugs ± SD	1.0 ± 1.3		0.9 ± 1.3		.64	.52	n.s.

EASI-severity > = 4, n (%)							
Physical health	20	(14.4)	21	(15.3)	4.4	.82	n.s.
Education, Work, Income	38	(28.0)	45	(33.9)	17.0	.02	< .05
Alcohol use	33	(23.4)	35	(25.2)	6.5	.60	n.s.
Drug use	135	(89.5)	122	(88.4)	10.6	.06	n.s.
Judicial	35	(25.0)	43	(11.8)	11.8	.11	n.s.
Relations	78	(56.5)	72	(52.5)	6.6	.47	n.s.
Psycho-emotional	71	(51.4)	72	(52.5)	2.0	.96	n.s.

Mean EASI Severity Indexes ± SD							
Physical health	2.0 ± 1.6		1.9 ± 1.6		.34	.73	n.s.
Education, Work, Income	2.5 ± 1.5		2.5 ± 1.8		.13	.90	n.s.
Alcohol	2.3 ± 1.7		2.2 ± 1.9		.47	.64	n.s.
Drugs	5.0 ± 1.1		4.6 ± 1.1		3.1	.002	< .05
Legal	2.4 ± 1.7		2.4 ± 2.0		-.09	.93	n.s.
Family	3.8 ± 1.5		3.6 ± 1.6		.85	.40	n.s.
Psycho-emotional	3.5 ± 1.8		3.4 ± 1.9		.76	.90	n.s.

EASI: EuropASI; n.s.: not significant; EASI-severity > = 4 means that treatment is needed [46]

### Adherence to treatment

The primary outcome measure for this study was treatment adherence at and beyond eight sessions. The results confirmed enhanced likelihood of this desired effect in patients allocated to the experimental condition.

Table 3 showed that adherence to treatment at and beyond eight sessions improved statistically significant in individual treatment where assessment and feedback was given (RR = 1.6,95%CI:1.2-2.1 in intention-to-treat (n = 227) or RR = 1.6,95%CI:1.2-2.2 in per-protocol (n = 211)). It means that 60% more persons adhered to individual treatment for at least eight sessions if continued assessment and feedback was provided within the individual treatment sessions, compared to those who received regular sessions. This result was found in both intention-to-treat and per-protocol analysis.

**Table 3:** Adherence at and beyond eight and twelve sessions in intention-to-treat and per-protocol analysis

	> = 8 sessions	> = 12 sessions
Intention-to-treat (n = 227)		
experimental	53.4%	33.6%
control	34.2%	20.7%

Risk ratio (RR)	1.6	1.6
95%CI	1.2-2.1	1.0-2.5

Per-protocol (n = 211)		
experimental	56.0%	36.0%
control	34.2%	20.7%

Risk ratio (RR)	1.6	1.7
95%CI	1.2-2.2	1.1-2.7

A second outcome measure was also set for adherence to treatment at and beyond twelve sessions. Yet, 90% of the assessment and feedback sessions (Table 1) took place within the first twelve sessions. In intention-to-treat analysis, the results showed again statistically significant improved adherence at and beyond twelve sessions, where continued assessment and feedback took place (RR = 1.6, 95%CI:1.0-2.5, n = 227). In per-protocol analysis, the results even showed 70% more persons that adhered to individual treatment for at least twelve sessions (RR = 1.7, 95%CI:1.1-2.7, n = 211).

## Discussion

Adherence in addiction treatment and studies is an important issue [40]. The results indicate that counselling sessions which included assessment with feedback directly to patients increased adherence to outpatient session-based treatment of substance use disorder, compared to an approach without assessment with feedback. Our results can be compared with findings in general health care [11] and in psychotherapy [41], although not 1:1. The nature of this trial was different in several aspects: (1) structuring treatment and continuing assessment was part of the intervention itself; (2) the trial - a non-medical intervention - was conducted in a population with multiple substance use disorder, where drop-out with low adherence and attendance rates are common [42], and (3) the design provided the inclusion of experienced therapists themselves to realise the intervention.

As a consequence, this study had limitations. Conducting a well-structured randomized controlled trial in a natural setting, where no manuals or protocols are used, does limit the conditions in the control group, because of possible contamination [15,14]. To keep the design pure, further structuring and assessments in the control group were not designated. Therefore, the primary outcome measure was limited to adherence at and beyond eight sessions and no other outcome measures were included. Studies in psychotherapy have found that 25% of patients reach clinically significant change in subjective discomfort, interpersonal relationships and social role functioning after five sessions, 50% reach clinically significant change after nine sessions, and 75% do so after 17 sessions [43]. In this study, most of the benefit was expected next to the first seven sessions, during which patients in the experiment were exposed to the assessment with feedback sessions. Application of a cut-point at twelve sessions also showed significant differences between the control group and the experimental group.

The strengths of this trial included its being built upon an already existing monitoring system, the use of well evaluated instruments and that it is associated with the real-world context where the intervention took place. Other than establishing protocols and providing a manual and continued training and supervision, the researcher had no influence on common processes in each centre in terms of managing capacity limitations on the unpredictable behaviour of patients. In all likelihood, the realised randomization and the participation level in a natural setting was the highest that could be reached in a trial where the trial's focus itself could lead to an increased workload without the benefit of additional clinical staff [40]. Moreover, the intervention took place within a real clinician-patient relationship, where good communication should consist of both instrumental (here, the trial intervention), and affective behaviours. Both elements, although unfavourable to meeting the internal validity benchmark, certainly enhanced external validity, and thus contributed to contextual evidence, favouring the probability of adoption in clinical settings [44,45]. The results may also have significance in the broader area of health care, where low compliance rates are of concern in case of preventive advice and/or chronic conditions to cope with.

## Conclusion

The use of assessment instruments with feedback directly to patients provided evidence to enhance adherence in routine practice in the treatment of substance use disorder. This finding may inspire the broader field of health care, especially in the care of chronic conditions and mental health to further elaborate continued measurement and outcome-feedback in daily practice. More research is needed to evaluate progress in social functioning and motivation to change in the treatment of substance use disorder, thereby using objective measures.

## Competing interests

The authors declare that they have no competing interests.

## Authors' contributions

VR and CAJDJ designed the study and wrote the protocol. VR managed the literature searches and summaries of previous related work. VR and DDB undertook the statistical analysis and interpretation. VR wrote the first draft of the manuscript. CAJDJ, EB and JDM supervised the whole project. All authors contributed to and have approved the final manuscript.

## Pre-publication history

The pre-publication history for this paper can be accessed here:

http://www.biomedcentral.com/1472-6963/11/123/prepub

## References

[B1] Leshner A Science is revolutionizing our view of addiction and what to do about it FOCUS The Journal of Lifelong Learning in Psychiatry 2003 I 2 194 195

[B2] Mc Lellan A Have we evaluated addiction treatment correctly? Implications from a chronic care perspective. Editorial Addiction 2002 97 249 252 10.1046/j.1360-0443.2002.00127.x 11964098

[B3] Mc Lellan A Lewis D O'Brien CP Kleber H Drug dependence, a chronic medical illness. Implications for treatment, insurance, and outcomes evaluation JAMA 2000 284 13 1689 1695 10.1001/jama.284.13.1689 11015800

[B4] O'Brien C Mc Lellan A Myths about the treatment of addiction Lancet 1996 347 237 240 10.1016/S0140-6736(96)90409-2 8551886

[B5] De Leon G Fixed and dynamic predictors of client retention in therapeutic communities J Subst Abuse Treat 1993 10 11 16 10.1016/0740-5472(93)90093-H 8383775

[B6] Kooyman M The therapeutic community for addicts. Intimacy, parent involvement and treatment success. 1993 Amsterdam: Swets & Zeitlinger

[B7] Soyez V De Leon G Rosseel Y Broekaert E The impact of a social network intervention on retention in Belgian therapeutic communities: a quasi-experimental study Addiction 2006 101 1027 1034 10.1111/j.1360-0443.2006.01441.x 16771894

[B8] Matto H Miller K Spera C Examining the relative importance of social context referents in predicting intention to change substance abuse behavior using EASE Addict Behav 2007 32 1826 1834 10.1016/j.addbeh.2006.12.015 17240077

[B9] De Leon G Hawke J Jainchill N Melnick G Therapeutic communities - enhancing retention in treatment using 'senior professor' staff J Subst Abuse Treat 2000 19 375 382 10.1016/S0740-5472(00)00124-0 11166502

[B10] Broekaert E What future for the therapeutic community in the field of addiction? A view from Europe Addiction 2006 101 1677 1678 17156161 10.1111/j.1360-0443.2006.01646.x

[B11] Greenhalgh J Meadows K The effectiveness of the use of patient-based measures of health in routine practice in improving the process and outcomes of patient care: a literature overview Journal of evaluation in clinical practice 1999 5 4 401 416 10.1046/j.1365-2753.1999.00209.x 10579704

[B12] Lambert M Hansen N Finch A Patient-focused Research: Using Patient Outcome Data to Enhance Treatment Effects J Consult Clin Psychol 2001 69 2 159 172 11393594

[B13] Knaup C Koesters M Schoefer D Becker T Puschner B Effect of feedback of treatment outcome in specialist mental healthcare: meta-analysis The British Journal of Psychiatry 2009 195 15 22 10.1192/bjp.bp.108.053967 19567889

[B14] Del Boca F Darkes J Enhancing the validity and utility of randomized clinical trials in addictions treatment research: II. Participant samples and and assessment Addiction 2007 102 1194 1203 10.1111/j.1360-0443.2007.01863.x 17511752

[B15] Kypri K Langley JD Saunders JB Cashell-Smith ML Assessment may conceal therapeutic benefit: findings from randomized controlled trial for hazardous drinking Addiction 2006 102 62 70 17207124 10.1111/j.1360-0443.2006.01632.x

[B16] Whipple J Lambert M Vermeersch D Smart D Nielsen S Hawkins E Improving the Effects of Psychotherapy: The Use of Early Identification of Treatment Failure and Problems-Solving Strategies in Routine Practice J Counselling Psychol 2003 50 1 59 68

[B17] Hawkins E Baer J Kivlahan D Concurrent monitoring of psychological distress and satisfaction measures as predictors of addiction treatment retention J Subst Abuse Treat 2008 35 207 216 10.1016/j.jsat.2007.10.001 18082998

[B18] Pope K Responsibilities in providing psychological test feedback to clients Psychological Assessment 1992 4 3 268 271

[B19] Clair D Prendergast D Brief psychotherapy and psychological assessments: entering a relationship, establishing a focus, and providing feedback Professional psychology: Research and Practice 1994 25 1 46 49

[B20] Finn S Tonsager M Therapeutic effects of providing MMPI-2 test feedback to college students awaiting therapy Psychological Assessment 1992 4 278 287

[B21] Altman D Schulz K Moher D Egger M Davidoff F Elbourne D Gotzsche P Lang T The revised CONSORT Statement for reporting randomized trials: explanation and elaboration Ann Intern Med 2001 134 8 663 694 11304107 10.7326/0003-4819-134-8-200104170-00012

[B22] Bennett J The consolidated standards of reporting trials (CONSORT). Guidelines for reporting randomized trials Nurs Res 2005 54 2 128 132 15778654 10.1097/00006199-200503000-00007

[B23] Schulz K Altman D Moher D CONSORT 2010 Statement: updated guidelines for reporting parallel group randomised trials Ital J Public Health 2010 7 3 325 332

[B24] Jadad A Randomised Controlled Trials. A user's guide 1998 Plymouth: Latimer Trend & Company Ltd21592376

[B25] Zelen M A new design for randomized clinical trials N Engl J Med 1979 300 1242 1245 10.1056/NEJM197905313002203 431682

[B26] Zelen M Randomized consent designs for clinical trials: an update Stat Med 1990 9 645 656 10.1002/sim.4780090611 2218168

[B27] Adamson J Cockayne S Puffer S Torgerson D Review of randomised trials using the post-randomised consent (Zelen's) design Contemp Clin Trials 2006 27 305 319 10.1016/j.cct.2005.11.003 16455306

[B28] Kokkevi A Hartgers C European adaptation of a multidimensional assessment instrument for drug and alcohol dependents Eur Addict Res 1995 1 208 210 10.1159/000259089

[B29] Hendriks V Kaplan C Van-Limbeek J Geerlings P The Addiction Severity Index: Reliability and validity in a Dutch addict population J Subst Abuse Treat 1989 6 133 141 10.1016/0740-5472(89)90041-X 2746712

[B30] Raes V Lombaert G EuropASI: A standard in De Sleutel, Belgium J Subst Use 2004 9 3-4 196 204 10.1080/14659890410001697514

[B31] Rollnick S Heather N Gold R Hall W Development of a short 'readiness to change' questionnaire for use in brief, opportunistic interventions among excessive drinkers Br J Addict 1992 87 743 754 10.1111/j.1360-0443.1992.tb02720.x 1591525

[B32] De Fuentes-Merillas L de Jong C Schippers G Reliability and validity of the Dutch version of the Readiness to Change questionnaire Alcohol Alcohol 2000 37 93 99 10.1093/alcalc/37.1.93 11825864

[B33] Raes V De Weerdt-Van Oene G Velasquez M De Maeseneer J de Jong C The use of RCQ-D in patients with poly drug abuse Addict Res Theory 2010 18 4 409 420 10.3109/16066350903291066

[B34] Küfner H Coenen M Indlekofer W PREDI Psychosoziale ressourcenorientierte Diagnostik. Ein problem- und lösungsorientierter Ansatz. [PREDI psycho-social resources oriented diagnostic system. A problem and solution oriented approach]. Version 3.0 2006 Lengerich: Pabst Science Publishers

[B35] Hunt S Alonso J Bucquet D Niero M Wiklund I McKenna S Cross cultural adaptation of health measures Health Policy 1991 19 33 44 10.1016/0168-8510(91)90072-6 10117390

[B36] Raes V Vanderplasschen W De Wilde M Delputte S Soyez V Broekaert E Measuring personal resources in a therapeutic community based treatment system: a reliability and validity study of the Dutch version of PREDI-Short Diagnosis Ther Commun J 2009 30 4 344 365

[B37] Del Boca F Darkes J Enhancing the validity and utility of randomized clinical trials in addictions treatment research: I. Treatment implementation and research design Addiction 2007 102 1047 1056 10.1111/j.1360-0443.2007.01862.x 17567393

[B38] Velasquez M Maurer G Crouch C Diclemente C Group treatment for substance abuse: a stages-of-change therapy manual [Dutch Translation by de Jong, CAJ & Verbrugge, CAG] 2006 Amsterdam: Harcourt Assessment

[B39] Lemeshow S Hosmer D Klar J Lwanga S Adequacy of Sample Size in Health Studies 1990 Chichester: Published on behalf of the World Health Organisation by John Wiley & Sons

[B40] McCarty D Gustafson D Wisdom J Ford J Choi D Molfenter T Capoccia V Cotter F The network for the Improvement of Addiction Treatment (NIATx): Enhancing access and retention Drug Alcohol Depend 2007 88 138 145 10.1016/j.drugalcdep.2006.10.009 17129680 PMC1896099

[B41] Hilsenroth M Cromer T Practice review: Clinician interventions related to alliance during the initial interview and psychological assessment Psychotherapy: theory, research, practice, training 2007 44 2 205 218 10.1037/0033-3204.44.2.205 22122211

[B42] Lefforge N Donohue B Strada M Improving session attendance in mental health and substance abuse settings: a review of controlled studies Behavior Therapy 2007 38 1 22 10.1016/j.beth.2006.02.009 17292691

[B43] Anderson E Lambert M A survival analysis of clinically significant change in outpatient psychotherapy J Clin Psychol 2001 57 7 875 888 10.1002/jclp.1056 11406801

[B44] De Maeseneer J Van Driel M Green L Van Weel C Research into practice II. The need for research in primary care The Lancet 2003 362 1314 1319 10.1016/S0140-6736(03)14576-X 14575979

[B45] Del Boca F Darkes J Enhancing the validity and utility of randomized clinical trials in addictions treatment research: III. Data processing and statistical analysis Addiction 2007 102 1356 1364 10.1111/j.1360-0443.2007.01864.x 17511751

[B46] Blanken P Hendriks V Pozzi G Tempesta E Hartgers C Koeter M Fahrner E Gsellhofer B Küfner H Kokkevi A European Addiction Severity Index EuropASI. Guide for training and administering the EuropASI interview 1996 European Commission: COST-A6 Working Group 4

